# First Report of *Haemaphysalis longicornis* (Neumann) in Oklahoma, USA

**DOI:** 10.3390/pathogens13100861

**Published:** 2024-10-02

**Authors:** Sarah A. Myers, Ruth C. Scimeca

**Affiliations:** Department of Veterinary Pathobiology, College of Veterinary Medicine, Oklahoma State University, Stillwater, OK 74078, USA; sarah.myers11@okstate.edu

**Keywords:** *Haemaphysalis longicornis*, cattle, *Theileria orientalis*, invasive species, range expansion, Oklahoma

## Abstract

*Haemaphysalis longicornis* (Neumann), the Asian longhorned tick, is a species native to East Asia, but invasive to Australia, New Zealand, and most recently, the United States. It has spread rapidly across the eastern United States after being established in New Jersey in 2017. Aiding this rapid expansion is the ability of this tick to reproduce parthenogenically and feed on diverse host species. In cattle, this tick can cause heavy burdens and act as a vector for the pathogenic hemoprotozoan parasite *Theileria orientalis*, genotype Ikeda, creating economic losses that impact the cattle industry. Here, we report Asian longhorned ticks, collected from cattle, a dog, and pastures and morphologically identified at the Oklahoma Animal Disease Diagnostic Laboratory as *H. longicornis* before molecular confirmation through PCR amplification of the *cox1* gene. Blood samples from infested cattle were collected and assessed molecularly for the presence of *T. orientalis*, with no pathogenic DNA detected. This report describes the first record of *H. longicornis* in Oklahoma and the farthest westward detection of this tick in the United States to date.

## 1. Introduction

Detection of *Haemaphysalis longicornis* (Neumann, 1901) in 2017 on an Icelandic sheep in New Jersey prompted widespread investigation of this invasive species, where a subsequent review of research collections revealed the earliest known non-quarantined introduction of this species to the United States occurred in 2010 [1,2]. Since 2017, surveillance predominantly in the eastern United States has detected *H. longicornis* in 20 states, including Washington D.C. [3]. Prior to the present report, this tick has been detected as far west as Arkansas and as far south as Georgia; as such, it is primarily distributed within the east and northeastern regions of the United States [3]. Asian longhorned ticks follow a three-host life cycle and have been collected from a wide variety of mammal and avian hosts in the United States, including companion animals, livestock, wildlife, and humans. Heavy infestation burdens have been documented on multiple species, notably cattle and white-tailed deer (*Odocoileus virginianus*) [4]. Host-seeking ticks have been frequently collected from the environment within the United States, including in both grassy pastures and wooded areas [4].

The rapid range expansion of this tick following its reported introduction is accelerated by multiple factors, including the species’ unique parthenogenic characteristic, ability to feed on a variety of host species, and survivability in a range of environmental conditions [5,6,7]. Some populations of *H. longicornis* have been found to tolerate temperatures between −2 °C and 40 °C and can be found in multiple habitats including pastures and mixed hardwood forests [8,9].

Molecular investigation of specimens collected in the United States revealed a closer genetic consistency with parthenogenic populations rather than bisexual populations of *H. longicornis* in other regions of the world, which is supported by the general lack of male specimens found in the US [5]. In places where parthenogenic ticks predominate, triploid females can reproduce without the presence of males by producing genetic clones, quickly building dense focal populations [10]. Haplotyping based on sequences of the mitochondrial *cox1* gene reveals that the Asian longhorned tick invasion of the United States originated from East Asian populations rather than Australian populations [5,11]. Ticks found within the US since 2017 are consistent with haplotypes H1, H2, and H3, which are commonly found in China and indicate the potential for three separate introductions into the country [5].

Worldwide, *Haemaphysalis longicornis* is traditionally considered a nuisance to livestock, and the major vector for the hemoparasite *Theileria orientalis*. Currently, eleven genotypes of *T. orientalis* have been identified according to variability in the major piroplasm surface protein (MPSP) [12,13]. Before the introduction of *H. longicornis* to the US, several *T. orientalis* genotypes had been identified, including Chitose and Buffeli, both usually considered non-pathogenic [14,15]. However, after detecting *H. longicornis* in the US, an increase in clinical bovine theileriosis cases has been reported [15,16,17]. This bloodborne parasite is transmitted to cattle mainly during tick feeding, where the organism infects erythrocytes and leukocytes, resulting in anemia, pyrexia, jaundice, weakness, and potentially death [18]. Infected cattle have been reported primarily in Virginia and West Virginia, where cattle populations are less dense than in South Central states, such as Oklahoma [16,19]. As this tick expands into areas with higher livestock densities, the impact on the health of the agricultural industry due to the presence of this tick and pathogen may become more prominent. 

In other countries, Asian longhorned ticks can transmit numerous bacterial, protozoal, and viral pathogens of veterinary and medical importance [20]. Concern within the United States regarding *H. longicornis* as a potential vector for numerous endemic pathogens of public health and veterinary importance has arisen as their range continues to expand into regions where these pathogens are vectored by other tick species. Multiple pathogens endemic to the United States have been detected in *H. longicornis*, including *Anaplasma phagocytophilum, Ehrlichia* spp., and *Rickettsia* spp., although vector competency has been largely undetermined for many of these pathogens [21,22,23]. Acquisition of *Rickettsia rickettsii* by co-fed *H. longicornis* has been observed experimentally, but evidence of natural acquisition or transmission has not been shown [24].

The present report documents the first collection of *H. longicornis* within the state of Oklahoma and identifies the first counties with established populations of this species in the state. This report will provide further information regarding the source of collection and the potential impact of this species as its range expands into the South Central United States.

## 2. Materials and Methods

### 2.1. Ticks

#### 2.1.1. Tick Submission and Morphologic Identification

On 31 July 2024, a local veterinarian reported several heifers from Craig County, Oklahoma, with a heavy tick burden. The ticks appeared different from the species typically seen in the area before. A total of 13 engorged ticks removed from cattle were submitted to the Oklahoma Animal Disease Diagnostic lab for identification. A second tick submission on 9 August 2024, from the same ranch but in Mayes County, located adjacent to Craig County, OK, included ticks removed from cattle and a dog and passively collected from pasture (*N* = 20). Ticks were submitted in 2 individual tubes with 70% ethanol, but not individually identified by source. Morphologic identification was performed at the Oklahoma Animal Disease Diagnostic Laboratory through parasitology, using stereomicroscopy and dichotomous keys [25,26].

#### 2.1.2. DNA Extraction

A subset of identified ticks, consisting of 2 engorged specimens collected from cattle (labeled OK3, OK4) and 2 unfed environmentally collected specimens (labeled OK5, OK6) were individually washed in 3% hydrogen peroxide, 70% alcohol, and a phosphate-buffered solution (PBS) sequentially. Washed ticks were manually dissected by circumferentially incising the idiosoma and removing all internal contents using sterile scalpel blades before the extraction of total genomic material using the Quick-DNA Miniprep Plus Kit (Zymo Research, Irvine, CA, USA), following manufacturer instructions. An extraction control utilizing water was made to ensure no contamination occurred during DNA isolation. The remaining exoskeletons were stored in ethanol at 20 °C.

#### 2.1.3. Amplification of *cox1* Gene

Extracted genomic material was used to amplify a 710 bp fragment of the mitochondrial cytochrome c oxygenase gene (*cox1*) by PCR using primers LCO1490-5′-GGTCAACAAATCATAAAGATATTGG-3’ and HCO2198-5′-TAAACTTCAGGGTGACCAAAAAATCA-3′ which have been previously used for *cox1* haplotyping of *Haemaphysalis longicornis* [5,27]. Individual 50 µL reactions included 25 µL of GoTaq DNA polymerase (Promega, Madison, WI, USA), 1 µL of each primer, 1 µL of bovine serum albumin (BSA), 5 µL of sample DNA, and 17 µL of nuclease-free water. Thermocycler conditions were as follows: initial denaturation at 95 °C for 3 min, and then 40 cycles of denaturation at 95 °C for 30 s, annealing at 50 °C for 30 s, and extension at 72 °C for 1 min before a final extension at 72 °C for 7 min. Appropriate negative controls were used. All PCR products were resolved by gel electrophoresis in 1.5% agarose gels and observed using GelRed Nucleic Acid Stain (Biotum, Fremont, CA, USA). Positive amplicons were purified using the GeneJET genomic DNA purification kit (Thermo Fischer, Waltham, MA, USA) according to the manufacturer’s instructions before bidirectional Sanger sequencing at Oklahoma State University (Stillwater, OK, USA).

#### 2.1.4. Phylogenetic Analysis

The resulting sequence data were evaluated and aligned using Geneious Prime Software V.2024.05 (Dotmatics, Boston, MA, USA) to create 612 bp consensus sequences of each tick for species confirmation through comparison with previously published reference sequences of *H. longicornis,* accessible in the Nucleotide BLAST database (National Library of Medicine). Sequence data from the 4 extracted Oklahoma ticks, as well as reference sequences in GenBank representing the 3 known haplotypes of *H. longicornis* (accession numbers MT034323, MT034173, and MT034418) currently documented within the United States, were used to generate a Neighbor-Joining phylogenetic tree to visualize the haplotype grouping of the presently collected ticks. Sequences derived from ticks in the present study are available in the GenBank database under accession numbers PQ231312–PQ231315.

### 2.2. Theileria orientalis

Due to concerns for the presence of *T. orientalis*, genotype Ikeda, in Oklahoma cattle, screening for this protozoan parasite started in November 2022, when a cattle herd from Lincoln County, OK, reported animals having clinical signs similar to Anaplasma marginale, despite testing negative for this pathogen. Even though H. longicornis was not reported in 2022, the movement of animals and the lack of detection of ticks were initially a concern.

#### 2.2.1. Cattle Blood Samples

On 9 August 2024, a total of 10 blood samples from cattle originally identified as infested with *H. longicornis* were collected in EDTA tubes to be tested for *T. orientalis*.

Additionally, before the detection of *H. longicornis* in Oklahoma from November 2022 to June 2024, 179 blood samples from cattle in Oklahoma were tested for *T. orientalis* due to concerns about the presence of the Ikeda genotype in the area. 

#### 2.2.2. DNA Extraction

DNA was isolated from whole blood samples using the ReliaPrep Blood gDNA Miniprep System (Promega, Madison, WI, USA) according to the manufacturer’s instructions.

#### 2.2.3. Amplification of *MPSP* and *SSU rRNA* Genes

We amplified the *MPSP* by utilizing the primers MPSP-F (5′-CTTTGCCTAGGATACTTCCT-3′) and MPSP-R (5′-ACGGCAAGTGGTGAGAACT-3ing′) and utilizing the same conditions for the *H. longicornis cox1* PCR as described above but with a melting temperature of 57 °C for 1 min. Additionally, we amplified the small subunit (*SSU*) *rRNA* gene using primers SSU-F (5′-ATTGGAGGGCAAGTCTGGTG-3′) and SSU-R (5′-CTCTCGGCCAAGGATAAACTCG-3′) with the same conditions as for the *MPSP* gene [28,29]. Reactions were conducted on all 10 blood samples from Oklahoma cattle infested with *H. longicornis* and all 4 tick specimens extracted for the present report. Additionally, the same testing was performed on the 170 blood samples from Oklahoma cattle that were collected prior to detecting *H. longicornis* in the state. All positive amplicons were purified as described above and submitted for Sanger sequencing to Eurofins Genomics (Louisville, KY, USA). Due to the similarity of clinical signs observed in animals infected with *Anaplasma marginale* to those infected with *T. orientalis*, a PCR that amplifies a fragment of the *MSP4* gene was performed, utilizing the primers MSP45 (5′-GGGAGCTCCTATGAATTACAGAGAATTGTTTAC-3′) and MSP43 (5′-CCGGATCCTTAGCTGAACAGGAATCTTGC-3′) on all bovine blood samples as described by de la Fuente et al., 2003 [30].

## 3. Results

### 3.1. Tick Morphologic Identification

**Figure 1:**
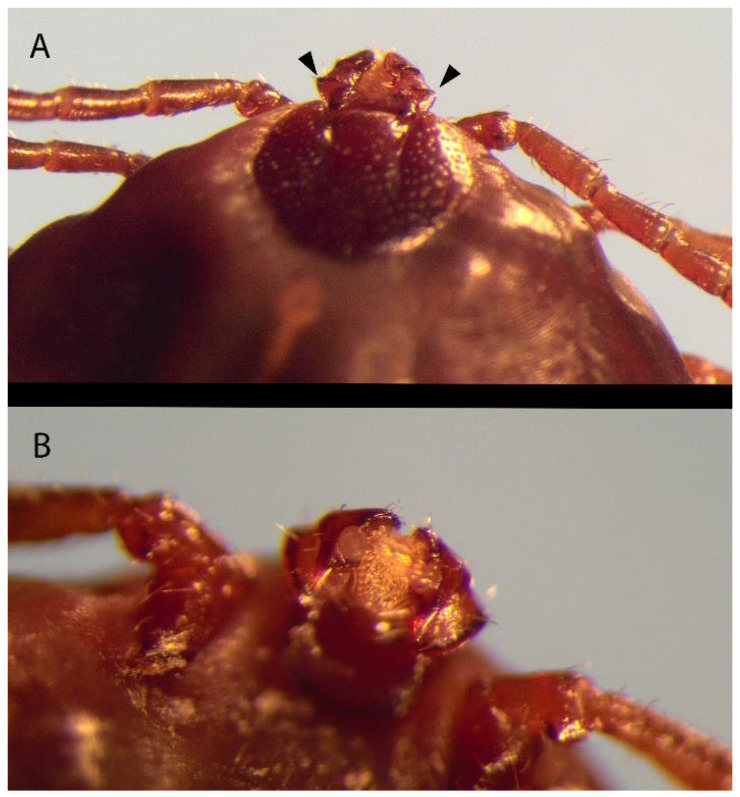
Dorsal (**A**) view and ventral (**B**) view of an engorged female *H. longicornis* identified after collection from Oklahoma cattle. The ticks pictured measure approximately 5 mm in width by 8 mm in length. The characteristic lateral projection on palpal segment 2 (black arrowheads) and a dorsal spur on palpal segment 3 can be seen.

**Figure 2:**
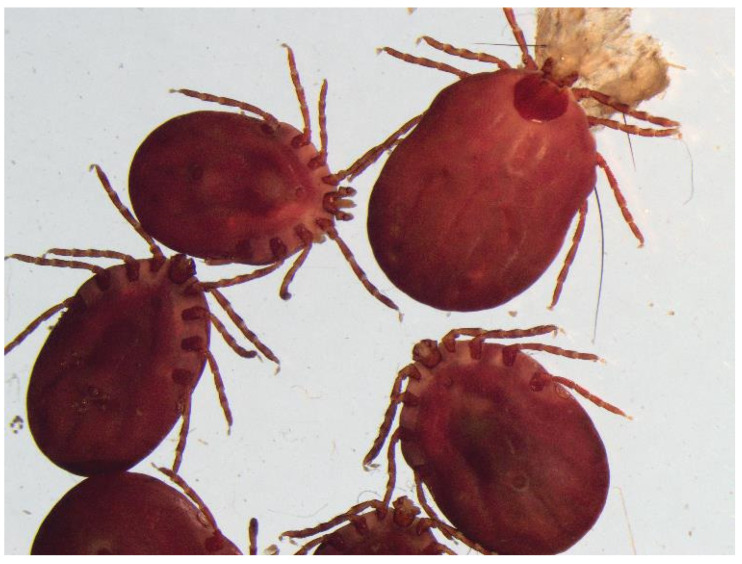
Multiple engorged female *H. longicornis* identified after collection from cattle residing in Oklahoma.

All ticks submitted were morphologically identified as *H. longicornis* adult females [25,26] (Figure 1 and Figure 2: images captured using the Olympus cellSens Entry imaging software version 3.2 and an Olympus model SZX2-ILLTQ microscope, Olympus Life Science, Waltham, MA, USA).

### 3.2. Tick Molecular Identification

**Figure 3:**
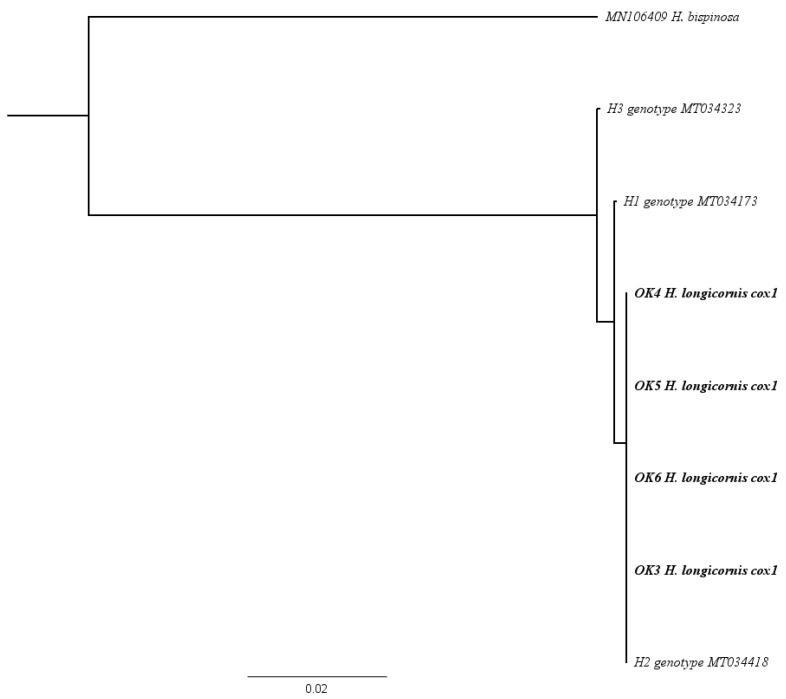
Neighbor-Joining phylogenetic tree depicting the relationship between *cox1* sequences of haplotypes previously documented in the United States (H1, H2, H3) and ticks collected for the present report (shown in bold), out-grouped to *Haemaphysalis bispinosa.* Ticks from this study are designated with OK3–OK6.

Genomic sequences derived from the four extracted ticks were 100% consistent with previously published *cox1* sequences of *H. longicornis* within the United States. All four extracted ticks from the present report were 100% identical to each other. Phylogenetic analysis based on a Neighbor-Joining phylogenetic tree and gene comparison using Nucleotide BLAST from the National Center for Biotechnology Information database (GenBank) determined all *cox1* sequences from the present report to be 100% identical to a previously submitted *cox1* sequence identified as haplotype 2 (MT034418), the predominant haplotype present in the United States (Figure 3). Only one or two base pairs differed between the Oklahoma ticks and genotypes H1 (MT034173) and H3 (MT03423).

### 3.3. Theileria orientalis

None of the blood samples collected from cattle (*N* = 10) infested with *H. longicornis* or individual *H. longicornis* ticks (*N* = 4) in Mayes and Craig counties, OK, were detected as infected with *T. orientalis.*

From the surveillance samples prior to the *H. longicornis* finding in Oklahoma, a total of 11/170 (6.1%) were positive for *T. orientalis*, genotype Chitose, and 6 were positive for *Anaplasma marginale* 6/170 (3.3%); GenBank accession numbers are PQ300672 and PQ301201 for the MPSP and SSU rRNA genes, respectively (Table 1).

## 4. Discussion

**Figure 4:**
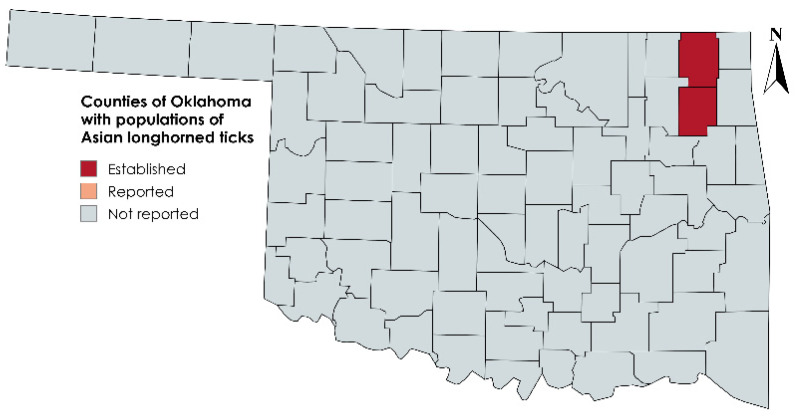
Map of Oklahoma depicting counties with recently established populations of Asian longhorned ticks. Established is defined as the identification of at least six individual ticks, or at least two of three host-seeking life stages within one calendar year. Reported is defined as the identification of less than six individual ticks of a single life stage within one calendar year [31].

In the present report, we describe the first discovery of *H. longicornis* parasitizing cattle in Oklahoma, collected in pastures where cattle reside. Despite active and ongoing surveillance efforts, the presence of *H. longicornis* in Oklahoma has not been previously described to the authors’ knowledge; the identification of these ticks confirms their presence in the state and extends the westward border of their known range within the United States. Based on the standards for establishing tick distributions and abundance set by the CDC, the ticks collected for the present report indicate that Craig and Mayes counties in northeast Oklahoma have an established population of *H. longicornis*, the first documented within the state (Figure 4) [31]. The presence of this tick in Oklahoma can be expected to have an impact on the cattle industry as well as on public health if it continues to expand within the state. 

Oklahoma continually ranks within the top five states for inventory of all cattle and calves within the United States, with a reported 4.60 million heads, representing 5.1% of all cattle in the country [32]. In numbers of beef cattle alone, Oklahoma ranks second in the country behind neighboring Texas. In addition to Oklahoma, the top four overall cattle-producing states include primarily those along historic cattle drives (i.e., Texas, Nebraska, and Kansas), many of which are threatened by the range expansion of this tick based on multiple predictive models [6,33]. The expansion of *H. longicornis* and *T. orientalis* Ikeda into the region threatens to burden the cattle industry by increasing animal mortality and decreasing overall production in areas with a density of animals not yet encountered within the current range of *H. longicornis* in the United States. States such as Virginia, which reports the highest number of counties with established *H. longicornis* populations at 39 counties and has documented cases of *T. orientalis* Ikeda, reports a 1.3 million head total of cattle and calves, less than half of that of Oklahoma [16,34]. Within the United States, limited data are available regarding the economic impact of *H. longicornis* and *T. orientalis*; however, marked economic loss has been reported in Asia, New Zealand, and Australia from clinical bovine theileriosis, with an estimated AUD 20 million annual loss reported in Australia alone [18,35].

Parasitism of cattle by ticks may cause physical damage, anemia, and “tick worrying”, resulting in detrimental irritation and unrest, and the transmission of pathogens [36]. These can result in the decreased production and well-being of animals and, in some cases, may result in death. Outbreaks of clinical theileriosis in beef and dairy cattle may result in significant losses to production and increased mortality [37,38]. Fatalities in US cattle that were heavily parasitized by *H. longicornis* have been described [1]. While *H. longicornis* has been determined to be an incompetent vector for *Babesia bovis*, a causative agent of Texas Cattle Fever, other pathogens including Heartland virus and Bourbon virus may be vectored by this tick, indicating the potential for an important One Health implication beyond the cattle industry [39,40,41]. Despite its association as a parasite of cattle, *H. longicornis* will readily feed on other domestic species and humans, so attention to potential human pathogens vectored by this tick is crucial. Outside of the United States, pathogens infecting other host species including *Babesia gibsoni* in dogs can be vectored by *H. longicornis*, indicating the potential for spreading within the United States with a competent vector present. However, experimental evidence has yet to be seen in US populations [42,43].

Management strategies on infested livestock operations in Tennessee that showed promise in reducing *H. longicornis* populations included implementing active surveillance methods, appropriate use of on-animal acaricides, pasture management, and maintenance of closed herds. Implementation of multiple of these strategies was found to have significantly reduced the questing of *H. longicornis* collected over 2 years [44]. Adoption of these strategies may be useful for Oklahoma livestock producers with the presence of *H. longicornis* populations. Animals with clinical signs consistent with anaplasmosis or theileriosis should be tested for both *A. marginale* and *T. orientalis* as the prevalence of this tick expands.

## 5. Conclusions

The present report describes the first case of *H. longicornis* in Oklahoma and identifies Craig County as the first county with an established population of these ticks within the state, followed by Mayes County. With the anticipated spreading of this tick in the state, continued active tick surveillance and testing of identified ticks and cattle for *T. orientalis* remains a priority. Increasing public awareness about *H. longicornis* and the potential pathogens it may transmit to people, pets, and livestock is important to reduce the threat to public health and animal husbandry in Oklahoma.

## Figures and Tables

**Table 1:** Number of bovine blood samples tested positive for *Anaplasma marginale* or *Theileria orientalis* by PCR according to Oklahoma county and year of collection.

Year Collected	County (OK)	*A.marginale* PCR Positive	*T. orientalis* PCR Positive *	Total Tested
2022	Atoka	0	3	61
2023	Creek	0	0	4
2022–2023	Lincoln	0	4	49
2024	Mayes	0	0	10
2022–2023	Okfuskee	0	4	43
2022	Pawnee	1	0	1
2022	Payne	4	0	10
2024	Pushmataha	0	0	1
2022	Washington	1	0	1

* *Theileria orientalis* genotype Chitose detected.

## Data Availability

Data from this study are freely available within the GenBank database under accession numbers PQ231312–PQ231315; PQ300672; and PQ301201.
